# Cinnamic acid promotes elongation of hair peg-like sprouting in hair follicle organoids via oxytocin receptor activation

**DOI:** 10.1038/s41598-024-55377-y

**Published:** 2024-02-27

**Authors:** Tatsuto Kageyama, Jieun Seo, Lei Yan, Junji Fukuda

**Affiliations:** 1 Kanagawa Institute of Industrial Science and Technology, 3-2-1 Sakado Takatsu-ku, Kawasaki, Kanagawa 213-0012 Japan; 2 Faculty of Engineering, Yokohama National University, 79-5 Tokiwadai, Hodogaya-ku, Yokohama, Kanagawa 240-8501 Japan; 3 Institute of Advanced Sciences, Yokohama National University, 79-5 Tokiwadai, Hodogaya-ku, Yokohama, Kanagawa 240-8501 Japan

**Keywords:** Cinnamic acid, Oxytocin receptor, Hair growth, Hair follicle organoid, Dermal papilla cells, Hair follicle, Biomaterials, Tissue engineering

## Abstract

Considerable global demand exists for the development of novel drugs for the treatment of alopecia. A recent report demonstrated that oxytocin promotes hair growth activity in human dermal papilla (DP) cells; however, its application in drugs or cosmetic products is challenging because rapid degradation and relatively large molecular weight prevent long-term topical administration on the scalp. Here, we examined cinnamic acid, a small molecule activator for oxytocin receptor (OXTR) expression. Treatment with cinnamic acid led to upregulation of OXTR and trichogenic gene expression in human DP cells. Furthermore, inhibition of OXTR with an antagonist, L-371,257, suppressed hair growth-related gene expression in DP cells. These findings suggest that cinnamic acid enhances the hair growth ability of DP cells via oxytocin signaling. Additionally, we tested the hair growth-promoting effects of cinnamic acid using hair follicle organoids in vitro and observed that cinnamic acid significantly promoted the growth of hair peg-like sprouting. These promising results may be useful for developing hair growth-promoting products targeting oxytocin.

## Introduction

The hair follicle, a skin appendage, regulates body temperature and safeguards vulnerable areas. Hair follicle loss arise from multiple factors, including genetics, dietary habits, stress, and anticancer medication^1–3^. The aesthetics of hairstyle play a pivotal role in shaping how people are perceived by others and a considerable demand exists for interventions addressing hair loss. According to the latest data from the International Society of Hair Restoration Surgery (ISHRS), the number of individuals that received hair restoration procedures in 2021 was 2,221,191, which included 1,592,588 nonsurgical patients (e.g., drug treatment) and 628,604 surgical patients (autologous hair transplantation)^4^. Less invasive drug therapy is desirable for hair loss treatment, but faces several limitations stemming from side effects and drug efficacy^5^. Therefore, researchers are working to find more efficient drugs for the treatment of hair loss.

Recently, we reported oxytocin as a potential hair growth promoter^6^. The study demonstrated that oxytocin treatment upregulated hair growth-related genes in dermal papilla (DP) cells, thereby promoting hair growth in an in vitro hair follicle growth model. Transdermal absorption of chemical compounds is prevented at Mw > 500^7^. Considering that oxytocin is an oligopeptide and a relatively large molecule (Mw = 1007), smaller molecular weight alternatives to oxytocin are necessary to activate the oxytocin signal pathway by topical administration to the skin.

*Cinnamomum cassia* is a Chinese herb and flavoring agent with diverse biological activities^8^, which exhibits analgesic effects on colds and shoulder, back, and joint pain. Additionally, it has been utilized as a cosmetic ingredient, as a study determined that *Cinnamomum cassia* extract has anti-aging effects on human dermal fibroblasts^9^. Recently, it was discovered that the extract of *Cinnamomum cassia* upregulated oxytocin receptor (OXTR) expression in fibroblasts and increased tenascin X production, which enhanced skin elasticity^10^. Drawing inspiration from this recent study, we hypothesize that certain components of *Cinnamomum cassia* could potentially activate the oxytocin signaling pathway in DP cells and thereby simulate hair growth.

**Figure 1:**
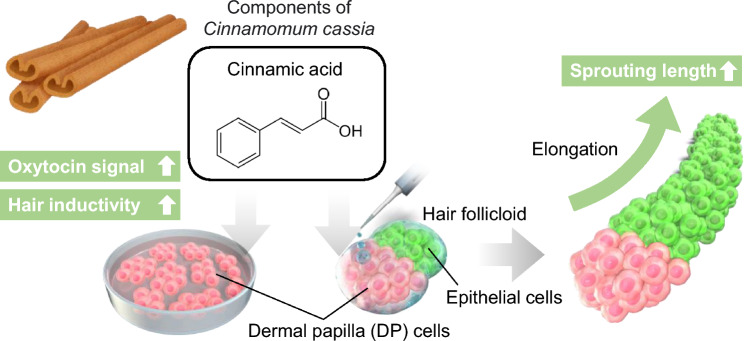
Schematic representation of experimental procedure. Human dermal papilla (DP) cells were seeded in 24-well plates and cultured in culture medium supplemented with cinnamic acid. Cinnamic acid treatment increased oxytocin signaling and hair inductivity. Cinnamic acid treatment increased the sprouting length of hair follicloids, suggesting that cinnamic acid promoted hair growth.

In this study, we assessed the impacts of cinnamic acid, a component of *Cinnamomum cassia* of small molecular weight (Mw = 148)*,* on the expression of OXTR and hair growth-related genes in DP cells (Fig. 1). Additionally, we examined the effects of cinnamic acid on hair growth using an in vitro hair follicle growth model. The resultant data has the potential to contribute to the advancement of novel hair loss treatments.

## Results

### Effects of cinnamic acid on DP cells

**Figure 2:**
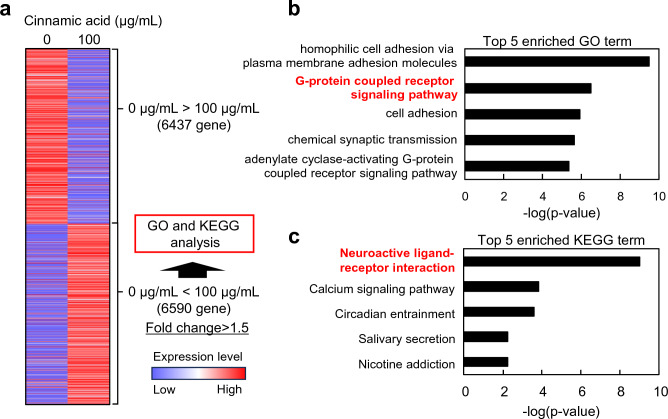
RNA-seq analysis of DP cells treated with cinnamic acid. (**a**) Heat map of differentially expressed genes (DEGs) between DP cells treated with/without cinnamic acid. (**b**) GO analysis; top five enriched pathways were identified by upregulated DEGs. (**c**) KEGG analysis; top five enriched pathways were identified by upregulated DEGs.

*Cinnamomum cassia* contains cinnamaldehyde and cinnamic acid, which are widely used in cosmetics. Therefore, we aimed to investigate the effects of these two components on human DP cells. However, experiments with cinnamaldehyde were challenging due to its low solubility in the culture medium, and in this study we focused on examining the effects of cinnamic acid. To identify the genes and signaling pathways affected by cinnamic acid treatment, we performed RNA-seq analysis. The analysis revealed that the addition of cinnamic acid resulted in the upregulation of 6590 genes (Fig. 2a). Further analysis using GO and KEGG pathway analyses revealed that the G-protein coupled receptor signaling pathway and neuroactive ligand–receptor interaction were among the top five terms associated with cinnamic acid treatment (Fig. 2b,c). Notably, OXTR genes, which are involved in these pathways, were upregulated through cinnamic acid treatment. The OXTR is a widely expressed G protein coupled receptor that binds its endogenous nonapeptide ligand oxytocin^11^. OXTR activation in the supraoptic nucleus plays a crucial role in regulating neurosteroid modulation of GABA-A receptors following childbirth^12^.

**Figure 3:**
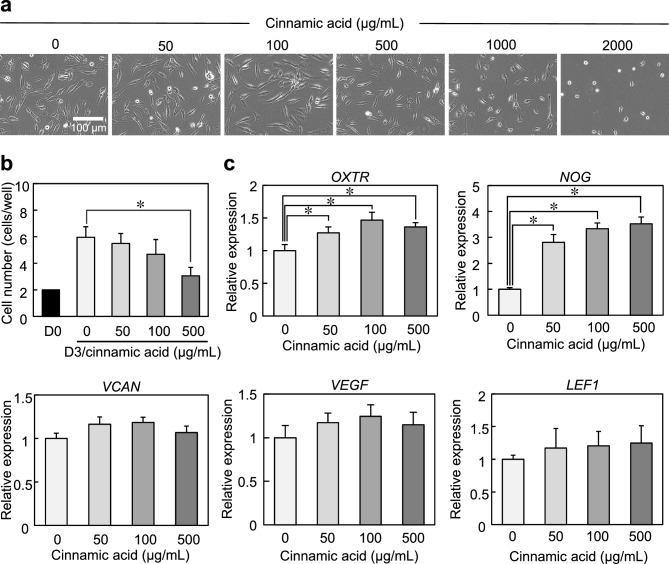
Effects of cinnamic acid treatment on DP cells. (**a**) Stereomicroscopic images of DP cells treated with cinnamic acid. (**b**) Number of proliferated cells after 3 days of culture. (**c**) Gene expression of OXTR and trichogenic markers at 3 days of culture. Error bars represent the standard error calculated from three independent experiments. Numerical variables were statistically evaluated using Tukey’s test. * indicates *p* < 0.05.

We evaluated the effects of cinnamic acid on the expression of OXTR and hair growth-related genes. DP cells were cultured for 3 days with a series of concentrations of cinnamic acid (ranging from 0 to 2000 μg/mL). At concentrations of 1000 and 2000 μg/mL, cinnamic acid induced cell damage, causing the detachment of cells from the dish (Fig. 3a). Additionally, cell proliferation was considerably inhibited at concentrations above 500 μg/mL, relative to the control condition of 0 μg/mL (Fig. 3b). At concentrations below 500 µg/mL, we observed a dose-dependent increase in the expression of OXTR and genes associated with hair growth, including noggin (NOG), versican (VCAN), vascular endothelial growth factor (VEGF), and lymphoid enhancer-binding factor-1 (LEF1), although no statistically significant differences in VEGF and LEF1 gene expression were observed (Fig. 3c).

**Figure 4:**
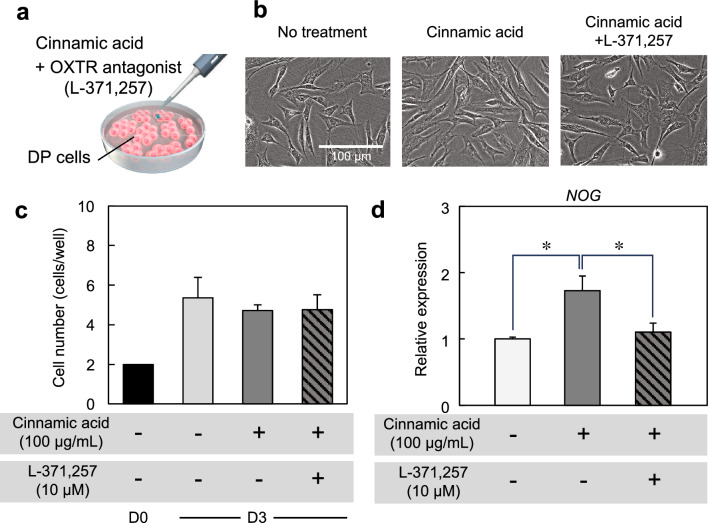
Treatment of OXTR antagonist and cinnamic acid on DP cells. (**a**) Procedures for OXTR inhibition in dermal papilla (DP) cells. (**b**) Stereomicroscopic images of DP cells treated with cinnamic acid and OXTR antagonist, L-371,257. (**c**) Number of proliferated cells after 3 days of culture. (**d**) Effects of OXTR inhibitor on a trichogenic marker expression in DP cells treated with cinnamic acid. Error bars represent the standard error calculated from three independent experiments. Numerical variables were statistically evaluated using Tukey’s test. * indicates *p* < 0.05.

To understand the relationship between OXTR and hair growth-related genes, DP cells were cultured in cinnamic acid-supplemented medium containing the OXTR antagonist L-371,257 (Fig. 4a). No differences in cell morphology and proliferation were observed among the three conditions (untreated control, cinnamic acid treatment, and cinnamic acid + L-371,257 treatment) (Fig. 4b,c). The expression of NOG decreased with the addition of L-371,257 (Fig. 4d). These results indicated that the upregulation of hair growth-related genes by cinnamic acid led through OXTR.

### Hair growth assay using hair follicloid

**Figure 5:**
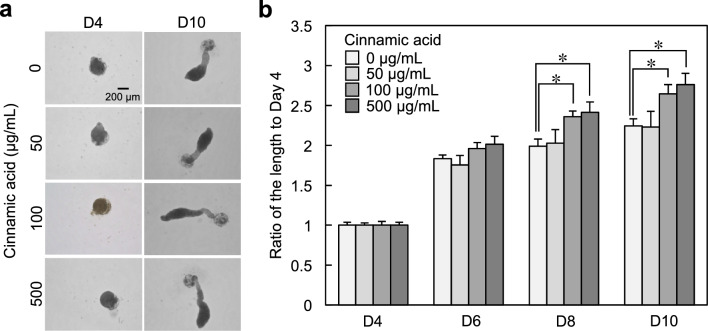
Hair growth testing using hair follicloids. (**a**) Microscope images of hair follicloids cultured with/without cinnamic acid for 10 days. Hair follicloids were permeabilized and observed using a stereomicroscope. (**b**) Length of sprouting structures with/without cinnamic acid. The graph shows the length ratio on days 6, 8, and 10 compared to that on day 4. We cultured at least 24 hair follicloids per treatment condition, which were used to calculate sprouting length. Numerical variables were statistically evaluated using Tukey’s test. * indicates *p* < 0.05.

We successfully developed a hair follicle organoid (hair follicloid) that could regenerate hair shafts in vitro with cells of mouse origin^13,14^. The application of this approach to cells of human origin showed that immature hair shaft-like structures elongated in response to minoxidil, a typical hair growth-promoting agent^15^. In our previous study, we examined potential hair growth-promoting drug candidates using human hair follicloids and identified the effects of oxytocin on hair growth^6^. In the present study, we evaluated the effects of cinnamic acid (0, 50, 100 and 500 μg/mL) on hair follicles using human hair follicloids. Cinnamic acid was added to the hair follicloids on day 4 of culture, and the medium was subsequently replaced with cinnamic acid-supplemented medium every 2 days for a duration of up to 10 days. The results showed a considerable increase in hair sprouting length on the 8th day of culture at a concentration of 100 and 500 μg/mL (Fig. 5a,b), suggesting that cinnamic acid exerts its hair-growing effect within the concentration range of 100–500 μg/mL.

## Discussion

*Cinnamomum cassia* is a tropical aromatic evergreen tree belonging to the *Camphoraceae* family, which is commonly used in traditional Chinese medicine. Moreover, revered as a traditional spice, its utilization transcends borders. In addition, *Cinnamomum cassia* extract has a wide range of pharmacological effects, including antitumor^16^, anti-inflammatory^17^, analgesic^18^, anti-obesity^19^, cardiovascular protective^20^, neuroprotective^21^, and anti-tyrosinase activity^22^. A recent study demonstrated that the application of *Cinnamomum cassia* extract to the skin of mice resulted in the promotion of hair growth^23^. These findings suggest that cinnamic acid, a component of *Cinnamomum cassia*, exhibits a hair growth-promoting effect, which is believed to be mediated through the upregulation of OXTR expression. The identification of cinnamic acid as a specific component with hair growth-promoting properties holds great promise for enhancing the effectiveness of hair growth products. In addition, the new understanding of the mechanism of hair growth-promoting effects mediated by oxytocin signaling will provide new insights by hair care science and help accelerate the search for new drugs targeting OXTR in the field of drug discovery.

The increase in cinnamic acid concentration decreased cell viability, which likely attributed to its acidifying effect on the pH of the culture medium, since obvious alteration in the phenol red color was observed in the medium. Higher concentrations of cinnamic acid may provide further effects by neutralizing the pH of culture medium.

Cinnamic acid caused a 1.25-fold increase in the elongation of hair sprouting length compared to the non-addition control in the hair growth assay using hair follicloids (Fig. 5B). In our previous study, oxytocin caused a 1.3-increase in the same assay^6^. These results suggest that cinnamic acid has almost the same level of hair growth-promoting effect as oxytocin. Oxytocin and cinnamic acid work through the same molecular mechanism of activating the oxytocin signaling pathway, but oxytocin binds to OXTR and cinnamic acid upregulates the expression of OXTR. Therefore, synergic effects of hair growth promotion can be expected by the combination of oxytocin and cinnamic acid. Oxytocin can be administered locally due to its short half-life (< 8 min) in blood^24^. However, due to its large molecular weight, its transdermal permeability is low, and approaches that stably activate the oxytocin signaling pathway are needed. Cinnamic acid is a stable, small molecule and has high transdermal permeability, and may reach other organs after application to the skin. Therefore, its side effects should be thoroughly investigated. Nevertheless, since both molecules are biomolecules safety concerns are expected to be low.

The most reliable in vitro model for evaluating hair growth-promoting effects is hair organ culture, in which human hair follicles are harvested from the scalp and cultured with drug candidates in vitro. However, the number of samples that can be harvested from humans is limited and the nature and hair cycle of individual hair follicles differ, making it challenging to obtain sufficient and reliable data. We developed an approach to prepare hair follicles in vitro (hair follicloids) that can regenerate hair shaft-like structures. Hair follicloids are prepared using cells isolated from multiple hair follicles, leading to a more uniform, prolific test system. Hair follicloids can be used to verify the efficacy of hair growth-promoting drugs by measuring the elongation of hair shaft-like structures, similar to conventional organ culture. In this study, we used hair follicloids to investigate the hair growth-promoting effects of cinnamic acid. However, in vivo studies are required to further investigate effects of transdermal dosing methods, concentrations, intervals, and side effects. In future, we plan to conduct such studies using mouse models of alopecia.

*Cinnamomum cassia* components and cinnamic acid derivatives have various functions. *Cinnamomum cassia* components (e.g. cinnamaldehyde, cinnamyl alcohol, and cinnamic acid) have a cinnamon scent and are used in cosmetics as fragrances ^25,26^. Ester derivatives of cinnamic acid (e.g. ethylhexyl methoxysilicate, isoamyl p-methoxysilicate, octocrylene, and cinoxate) have UV protective properties and are used in cosmetics as sunscreen^27^. In addition, cinnamic acid derivatives have antioxidant properties that may slow down the skin aging process^27^. Investigating the hair growth-promoting effects of these cinnamic acid derivatives may facilitate the development of a drug that combines the UV protection/anti-aging effects of hair follicles with hair growth-promoting effects. Notably, allergic dermatitis is one of the side effects of *Cinnamomum cassia* components and cinnamic acid derivatives. Cinnamaldehyde and cinnamyl alcohol are sensitizers of allergic dermatitis, thereby limiting their contents in cosmetics^28,29^. Therefore, the use of cinnamic acid derivatives necessitates consideration of adverse effects.

To the best of our knowledge, allergic effects have not been identified for the cinnamic acid used in this study. However, further studies are needed to confirm the side effects for the skin and other organs. Topical administration to the hair follicles may minimize side effects.

In conclusion, cinnamic acid activated DP cells and promoted hair growth in vitro via increase in OXTR expression. These findings encourage further research on the discovery of drugs targeting OXTR in patients with hair loss. Although the same cell source was used in this study, the efficacy of cinnamic acid should be evaluated in future using donor cells isolated from patients with multiple alopecias. In future studies, we intend to conduct a comprehensive analysis of gene expression using multiple donor cells from patients with alopecia.

## Materials and methods

### Preparation of human DP and epithelial cells

Adult human DP cells were obtained from PromoCell (Heidelberg, Germany), passaged up to passage 4 with dermal papilla cell growth medium (DPCGM; PromoCell), and used for 2D culture. Adult human follicular keratinocytes (epithelial cells) were obtained from the Scientific Cell Research Laboratories (Carlsbad, CA). DP cells at passage 4 and epithelial cells at passage 1 were used for organoid culture. Incubator gas tension was maintained at 21% O_2_ and 5% CO_2_ at 37 °C.

### Cinnamic acid treatment of DP cells

DP cells (2 × 10^4^ cells) were suspended in 0.5 mL DPCGM medium supplemented with 0, 50, 100, 500, 1000, or 2000 μg/mL Cinnamic acid (WAKO, Japan), and seeded into the wells of a 24-well cell culture plate (Corning Inc., Corning, NY, USA). Cells were counted using a cell counter (Chemometec, Denmark) after 3 min of trypsin–EDTA treatment. Gene expression in DP cells was assessed using real-time reverse transcription-polymerase chain reaction (RT-PCR) after 3 days of culture.

### Inhibition of OXTR in DP cells

DP cells (2 × 10^4^ cells) were suspended in 0.5 mL DPCGM medium supplemented with 100 μg/mL Cinnamic acid and 10 μM L-371,257 (MedChemExpress, NJ, USA), and seeded into the wells of a 24-well cell culture plate. Cells were counted using a cell counter (Chemometec, Denmark) after 3 min of trypsin–EDTA treatment. Gene expression in DP cells was assessed by RT-PCR after 3 days of culture.

### Cinnamic acid treatment of hair follicloids

To investigate the effects of cinnamic acid on hair growth, DP cells (5 × 10^3^ cells) and epithelial cells (5 × 10^3^ cells) were suspended in 0.2 mL advanced Dulbecco's Modified Eagle Medium/Nutrient Mixture F-12 (DMEM/F-12; Thermo Fisher Scientific) containing 2% (v/v) Matrigel (Corning Inc.) and seeded into the wells of a non-cell-adhesive round-bottom 96-well plate (Primesurface^®^ 96U plate; Sumitomo Bakelite Co., Ltd., Japan). DMEM/F-12 medium was supplemented with 10 μM cinnamic acid for 4–10 days after seeding. Then, 0.1 mL of the spent medium was replaced with fresh medium every 2 days. Hair sprout lengths were observed using an all-in-one fluorescence microscope (BZ-X810; Keyence).

### Gene expression analysis

Total RNA was extracted from the samples using RNeasy Mini Kit (Qiagen, Hilden, Germany) and used for complementary DNA synthesis using the ReverTra Ace^®^ RT-qPCR Kit (Toyobo, Osaka, Japan) according to the manufacturer’s instructions. Subsequent qRT-PCRs were performed using the StepOne Plus RT-PCR system (Applied Biosystems, Waltham, MA, USA) with SYBR^®^ Premix Ex Taq™ II (Takara Bio, Kusatsu, Japan) and primers for amplifying human OXTR, NOG, ALP, VEGF, LEF1, and glyceraldehyde-3-phosphate dehydrogenase (GAPDH). The primers used in this study are listed in Table 1. All gene expression levels were normalized to those of GAPDH. The 2^−∆∆Ct^ method was used to determine relative gene expression levels, and they were presented as the mean ± standard error of three independent experiments. Statistical evaluation of the numerical variables was conducted using Tukey’s test, with a *p* < 0.05 indicated statistical significance.

**Table 1:** PCR primer sequences.

Genes	Forward (5′–3′)	Reverse (5′–3′)
*OXTR*	CCTTCATCGTGTGCTGGACG	CTAGGAGCAGAGCACTTATG
*NOG*	CTGGTGGACCTCATCGAACA	CGTCTCGTTCAGATCCTTTTCCT
*ALP*	ATTGACCACGGGCACCAT	CTCCACCGCCTCATGCA
*VEGFA*	ACTTCTGGGCTGTTCTCG	TCCTCTTCCTTCTCTTCTTC
*LEF1*	CTTCCTTGGTGAACGAGTCTG	TCTGGATGCTTTCCGTCAT
*GAPDH*	TGGAAGGACTCATGACCACAG	GGATGATGTTCTGGAGAGCCC

### RNA-seq analysis

Total RNA was extracted from DP cells with or without 100 μg/mL cinnamic acid treatment for 3 days using an RNeasy Mini Kit (Qiagen). RNA-seq analysis was performed using Takara Bio. Significantly upregulated genes in DP cells subjected to OXTR treatment were used for gene ontology and Kyoto Encyclopedia of Genes and Genomes pathway analyses using the Database for Annotation, Visualization, and Integrated Discovery (http://david.abcc.ncifcrf.gov/)^30,31^.

### Statistical analysis

Statistical analyses of gene expression levels and hair sprout length were conducted using Tukey’s test, and the results were considered statistically significant at *p* < 0.05. All data are presented as mean ± standard error.

## Data Availability

The datasets generated and analyzed during the current study are available in the NCBI repository, GSE252536.

## References

[1] Thom E (2016). Stress and the hair growth cycle: Cortisol-induced hair growth disruption. J. Drugs Dermatol..

[2] Gokce N (2022). An overview of the genetic aspects of hair loss and its connection with nutrition. J. Prev. Med. Hyg..

[3] Saraswat N, Chopra A, Sood A, Kamboj P, Kumar S (2019). A descriptive study to analyze chemotherapy-induced hair loss and its psychosocial impact in adults: Our experience from a tertiary care hospital. Indian Dermatol. Online J..

[4] Relevant_Research. *International Society of Hair Restoration Surgery: 2022 Practice Census Results*. https://ishrs.org/wp-content/uploads/2022/04/Report-2022-ISHRS-Practice-Census_04-19-22-FINAL.pdf (2022).

[5] Nestor MS, Ablon G, Gade A, Han H, Fischer DL (2021). Treatment options for androgenetic alopecia: Efficacy, side effects, compliance, financial considerations, and ethics. J. Cosmet. Dermatol..

[6] Kageyama T, Seo J, Yan L, Fukuda J (2023). Effects of oxytocin on the hair growth ability of dermal papilla cells. Sci. Rep..

[7] Bos JD, Meinardi MM (2000). The 500 Dalton rule for the skin penetration of chemical compounds and drugs. Exp. Dermatol..

[8] Zhang C (2019). *Cinnamomum cassia* Presl: A review of its traditional uses, phytochemistry, pharmacology and toxicology. Molecules.

[9] Takasao N, Tsuji-Naito K, Ishikura S, Tamura A, Akagawa M (2012). Cinnamon extract promotes type I collagen biosynthesis via activation of IGF-I signaling in human dermal fibroblasts. J. Agric. Food Chem..

[10] POLA. *BA Grandluxe*. https://www.pola.com.hk/brand/ba/grandluxe/en/ (2019).

[11] Chini B, Verhage M, Grinevich V (2017). The action radius of oxytocin release in the mammalian CNS: From single vesicles to behavior. Trends Pharmacol. Sci..

[12] Tuem KB, Atey TM (2017). Neuroactive steroids: Receptor interactions and responses. Front. Neurol..

[13] Kageyama T (2022). Reprogramming of three-dimensional microenvironments for in vitro hair follicle induction. Sci. Adv..

[14] Kageyama T, Anakama R, Togashi H, Fukuda J (2022). Impacts of manipulating cell sorting on in vitro hair follicle regeneration. J. Biosci. Bioeng..

[15] Kageyama T, Miyata H, Seo J, Nanmo A, Fukuda J (2023). In vitro hair follicle growth model for drug testing. Sci. Rep..

[16] Eweys AS, Zhao YS, Darwesh OM (2022). Improving the antioxidant and anticancer potential of *Cinnamomum cassia* via fermentation with *Lactobacillus plantarum*. Biotechnol. Rep. (Amst.).

[17] Liao JC (2012). Anti-Inflammatory activities of *Cinnamomum cassia* constituents in vitro and in vivo. Evid. Based Complement Alternat. Med..

[18] Balayssac D (2015). Prevention of oxaliplatin-induced peripheral neuropathy by a polyamine-reduced diet-NEUROXAPOL: Protocol of a prospective, randomised, controlled, single-blind and monocentric trial. BMJ Open.

[19] Song MY (2017). *Cinnamomum cassia* prevents high-fat diet-induced obesity in mice through the increase of muscle energy. Am. J. Chin. Med..

[20] Kwon H (2015). Cinnamon and its components suppress vascular smooth muscle cell proliferation by up-regulating cyclin-dependent kinase inhibitors. Am. J. Chin. Med..

[21] Yu HS, Lee SY, Jang CG (2007). Involvement of 5-HT1A and GABAA receptors in the anxiolytic-like effects of *Cinnamomum cassia* in mice. Pharmacol. Biochem. Behav..

[22] Chang CT, Chang WL, Hsu JC, Shih Y, Chou ST (2013). Chemical composition and tyrosinase inhibitory activity of *Cinnamomum cassia* essential oil. Bot. Stud..

[23] Wen TC (2018). Effect of *Cinnamomum osmophloeum* kanehira leaf aqueous extract on dermal papilla cell proliferation and hair growth. Cell Transplant..

[24] Leng G, Sabatier N (2016). Measuring oxytocin and vasopressin: Bioassays, immunoassays and random numbers. J. Neuroendocrinol..

[25] Letizia CS, Cocchiara J, Lalko J, Lapczynski A, Api AM (2005). Fragrance material review on cinnamyl alcohol. Food Chem. Toxicol..

[26] Letizia CS, Cocchiara J, Lapczynski A, Lalko J, Api AM (2005). Fragrance material review on cinnamic acid. Food Chem. Toxicol..

[27] Gunia-Krzyzak A (2018). Cinnamic acid derivatives in cosmetics: Current use and future prospects. Int. J. Cosmet. Sci..

[28] Heisterberg MV, Menne T, Johansen JD (2011). Contact allergy to the 26 specific fragrance ingredients to be declared on cosmetic products in accordance with the EU cosmetics directive. Contact Dermatitis.

[29] Schnuch A, Uter W, Geier J, Gefeller O, Group IS (2002). Epidemiology of contact allergy: An estimation of morbidity employing the clinical epidemiology and drug-utilization research (CE-DUR) approach. Contact Dermatitis.

[30] Kanehisa M, Goto S (2000). KEGG: Kyoto encyclopedia of genes and genomes. Nucleic Acids Res..

[31] Kanehisa M, Furumichi M, Sato Y, Kawashima M, Ishiguro-Watanabe M (2023). KEGG for taxonomy-based analysis of pathways and genomes. Nucleic Acids Res..

